# Neural Alterations in Interpersonal Distance (IPD) Cognition and Its Correlation with IPD Behavior: A Systematic Review

**DOI:** 10.3390/brainsci11081015

**Published:** 2021-07-30

**Authors:** Xinxin Huang, Shin-Ichi Izumi

**Affiliations:** 1 Department of Physical Medicine and Rehabilitation, Tohoku University Graduate School of Medicine, Sendai 980-8575, Japan; huangxinxinpsy@yahoo.co.jp; 2 Department of Physical Medicine and Rehabilitation, Tohoku University Graduate School of Biomedical Engineering, Sendai 980-8575, Japan

**Keywords:** interpersonal distance, neural activation, functional connectivity, correlation

## Abstract

Background. Interpersonal distance (IPD) plays a critical role in a human being’s social life, especially during interpersonal interaction, and IPD is non-verbal social information and not only provides silent cues but also provides a secure space for personal relationships. IPD has been a research field of neural studies from the recent decade, researches had provided behavior and neural correlates of IPD. Objectives. This review aims to summarize the experimental paradigms of IPD-neural research, to reveal the neural activity processes associated with it, and to explore the correlation between IPD-neural activity and IPD-behavior. Methods. We conducted a standardized systematic review procedure, including the formal search method be adopted to seek out any type of studies related to IPD and brain, then devised them into categories to make a systematic review. Results. 17 articles met the inclusion criteria of the review, 5 event-related potential (ERP) studies measured the amplitude and latencies of ERPs, and 12 functional magnetic resonance imaging (fMRI) studies provided the neural activation during IPD tasks. In addition, the passive IPD experimental paradigm is the main experimental paradigm for exploring neural activity in IPD cognition, with the parietal lobe, motor areas, prefrontal lobe, and amygdala being the main brain areas involved. Functional connections between the identified brain regions were found and have a moderate correlation with IPD behavior. Conclusions. This review provides the neural activity of the IPD interaction process. However, the insufficient ecological validity of IPD tasks and ignore the initiative of people in IPD interaction. Therefore, there is a large research space on this topic. The work of the current systematic review contributed to linking the external performance and inner neural activities of IPD.

## 1. Introduction

Interpersonal distance (IPD), a silent space and interaction zone, is a physical distance that individuals choose to maintain between themselves and others while interacting [1]. IPD conveys non-verbal social information, like body gestures, facial expressions, and eye contact. As a kind of non-verbal communication, IPD has the same communication power as verbal in human social life, even it offers vital interpersonal and emotional information beyond language. Dynamic changes in IPD reveal information about changes in relationships between individuals. Narrowing IPD means a proximity tendency to the other one, while expanding IPD may symbolize being threatened [2]. IPD is closely related to personal space, and the size of personal space affects IPD. The protective response of personal space occurs when we are threatened and may manifest in the process of inter-individual interactions by expanding IPD, especially in social dysfunction groups [3]. Personal space is an important self-protective zone and defensive space. Although the establishment and adjustment of IPD are closely related to the size of personal space, IPD has a clear desire to establish social relationships and communication with others. We adjust the spatial distance between each other according to the social situation and our feelings. The proximity of IPD is influenced by the expression and understanding of the intentions of the person with whom the individual is interacting [4,5,6]. Moving closer is a message of intimacy needs. However, moving backward means the need for greater IPD or a response to self-protection. We deal with IPD with others and adjust it according to social interaction objects, which constitutes a significant and meaningful part of social life. As a member of society, keeping a proper IPD with members is an essential psychological function [7]. Maintaining a suitable IPD with others contribute to a smooth social communication process and the establishment and maintenance of good relationships. While, a far IPD from others is not easy to build up interpersonal relationships, or being too close, making others feel violated or be attacked [5,8,9]. Moreover, the interpersonal distance between individuals reflects the distance and quality of their social relationships [10]. The IPD interaction between persons essentially reflects the nature of social relationships, for example, we keep a closer IPD with a friend than that with a stranger. Edward T. Hall devised the IPD into four types base on social context and social relationship [11]. The IPD termed by Hall was the relative distances within other people. The largest distance is the public distance during public speaking, which ranges from more than 3.7 m to 7.6 m. Following the social distance between 1.2 m to 3.7 m, keeping from the people we know. And then the space within friends and families, from 0.46 m to 1.2 m. Last but not the least, intimate distance is shared for hugging or whispering in close relationships, less than 0.45 m. Therefore, we may know the relationship between two people bases on their IPD. That is, IPD plays as a reference for social relationships, especially when we interact with people from different cultural backgrounds [12].

As we mentioned before, IPD is one of the key psychological capabilities. IPD not only helps to establish interpersonal boundaries and private space but also guides us to create a good enough space for interaction with others. From the perspective of human development, IPD begins with the integration of mother and infants without distance. Infants and toddlers are embraced by their mothers, skin to skin, and they do not have the concept of personal boundaries and personal distance. They believe that they and their mother are one. As children grow up, they gradually learn that they are themselves and their mother is their mother. The growth is from the transitional space to the independent IPD. 

Thus, studies on IPD are important for understanding human social behavior and development. IPD is a complex social behavior, a body of researches has focused on this field. It was found in the early stages of IPD studies that demographic variables influence the IPD, the personal distance of men is larger than that of women [13,14,15], Taller people require larger IPD [16,17]. Furthermore, researchers turned to the influential factors of psychology. They found a high level of sensitivity [18], insecure attachment styles [19], post-traumatic stress disorder [20], eating disorders [21], loneliness [22,23], childhood abuse experience [24,25], peripersonal space representation [2,26], and aggressiveness [27] significantly increases the IPD. Additionally, researchers explored the impact of changes in external physical stimuli on the IPD. For example, listening to active music made people closer to others [28]. Compared to dark lighting conditions, a bright light condition reduced the space of interpersonal distance [29]. Additionally, Abnormal performance of IPD, associated with a variety of psycho-psychiatric disorders, such as autism, anorexia [21]. Besides, the changes in the IPD are also related to blood pressure [30]. Moreover, the study of the IPD plays an important role to understand behavior changes during the current COVID-19 pandemic, which means IPD changes are affected by COVID-19 [31]. During COVID-19, subjectively perceived COVID-19 risk and the related level of anxiety increases the IPD [32], wearing a mask was able to reduce IPD [33], while people still maintain a relatively far IPD from negative emotional people, despite the fact they are wearing masks [34]. Although there is a large number of studies on extrinsic behavioral changes in IPD, the intrinsic neural activity associated with IPD is still poorly explored.

With the evolution of cognitive neuroscience, neuroscientists paid attention to the neural correlates of IPD both in non-human and human areas in recent decades. From the view of space protection and survival, initial evidence of the neural basis of IPD was coming from animal experiments. Electrical stimulation of the ventral intraparietal area (VIP) and the precentral gyrus (PZ) in the monkey’s brain evoked spatial defense responses, such as head retraction and hand movement to the outer space [35]. It suggested that this area is related to protective personal space in animals. In addition, when a monkey watches a small ball space close to and away from the face (37.5 cm–2 cm), the neuron response of PZ increases as the distance decreases [36]. Though the primate experiment has provided evidence of neurological factors that may explain IPD, the mice experiment better explored the IPD neural activity of animals in social situations. The enhancement of single-neuron activity in the medial prefrontal cortex (mPFC) of mice was observed when approaching a stranger mouse in a modified version of the three-chamber test [37]. In recent three-chamber research, similar results are found. The activity from dopaminergic neurons of the ventral tegmental area was an increase when toward a juvenile mouse other than an object with social odor [38]. Thus, studies of animal experiments offer a favorable reference for exploring the IPD neural mechanism of human beings. 

The application of functional brain imaging and technology is crucial in the field of IPD as brain and behavioral changes are concomitant with each other. On the one hand, the study of the effects of brain lesions on IPD can be a reference for the neurophysiological basis of IPD. In an amygdala lesion case study of IPD, the patient keeps a close IPD during the stop-distance task, indicating the loss of IPD sense with the dysfunction of the amygdala [9]. That is, the amygdala is responsible for IPD modulation. Moreover, the orbitofrontal cortex (OFC) was found critical for maintaining appropriate interpersonal distance. Patients with OFC damage showed significantly closer distance compare to the healthy group and patients with dorsolateral prefrontal damage in the stop-distance task [39]. On the other hand, brain activities during IPD tasks have progressed in researches. Early N1 event-related potential (ERP) component was found in a computerized IPD task between stranger and friend conditions [40]. Furthermore, the dorsal intraparietal sulcus (DIPS) and the ventral premotor cortex (PMv) account for the approaching face, which stands for the IPD proximity, in healthy and Schizophrenic subjects [41,42]. However, the neural mechanisms underlying IPD are not well understood, as the study groups and task paradigms were varied according to the research objectives. It is important to further investigate the neural correlates of IPD, including the neural activation, functional connectivity, and the correlation between IPD behavior and neural activities. 

IPD is a complex and integrated social behavior, an in-depth understanding of the neural mechanisms associated with it is a vast and complicated task. How are the neural activity processes of IPD recorded and which brain regions are involved in the cognitive process of IPD? What are the connections between these brain regions? Are the neural IPD studies qualified to reflect the IPD behavioral processes? Hence, the current systematical review is to answer these questions. Besides, there were no articles that have systematically reviewed the neural mechanism of IPD. Thus, the present systematic review was focus on this topic, to sort out all the scientific publications related to neural correlates of IPD. This review introduced the experimental paradigms of IPD and explored the strengths and weaknesses between them. As well, this article focused on summarized the brain activation in IPD, functional connectivity, and correlation between IPD and neural activities. Furthermore, based on a systematic review of the researches, we discussed the limitations of previous studies and proposes the challenges and opportunities in the field of IPD neuroscience. This systematic review contributes to the construction of a standard, ecologically validated experimental research paradigm for IPD, helps to comprehend the neural basis of IPD in a multifaceted way, and provides a reference for subsequent studies. 

## 2. Materials and Methods

This systematic review was registered in INPLASY (registration number: INPLASY202170074); It was conducted and reported in accordance with the Preferred Reporting Items for Systematic Reviews and Meta-Analyses (PRISMA) Statement [43].

### 2.1. Eligibility Criteria

#### 2.1.1. Included Studies

This review aims to investigate the neural mechanisms related to IPD, eligible studies included those which conduct the IPD evaluation or cognition under neural methods, like functional magnetic resonance imaging (fMRI), ERP.

IPD studies mainly adopted within-subjects or between-within subjects design to ensure that factors like age, gender, culture, height, and personal character were equivalent across experimental conditions. Therefore, this review included any types of evidence that related to the IPD neural process.

#### 2.1.2. Included IPD Paradigms

IPD paradigm must have met the physical distance between subjects and others. In addition, the experimental procedure must include the process of interpersonal distance assessment or interpersonal distance recognition.

If the IPD paradigm of studies included components other than IPD cognition (e.g., relationship evaluation, peripersonal space evaluation) were excluded.

#### 2.1.3. Included Subjects

Eligible studies included healthy subjects who self-report or were screened by the researchers, without physical illnesses, mental disorders, psychiatric, or neurological.

Eligible studies also included patients (e.g., mental illness, brain lesion), to study the effect of brain function variation on IPD.

Animal studies were excluded.

#### 2.1.4. Included Results

The outcomes of the studies have to contain neural results, especially the functional neural changes during the IPD-tasks. The studies, which only collect the neural structure in the brain were excluded. Outcomes of neural alterations must be recorded through devices used in neuroscience research, such as fMRI and electroencephalograph (EEG).

### 2.2. Search Methods and Study Selection

Database searching was completed on 2 April 2021 and included searches of Ovid (Medline, Embase, Cochrane, PsycINFO). Publication dates or language were no a limitation for search.

The search terms were entered into the database as follows: (preferred social distance OR personal space OR interpersonal space OR social space OR personal distance OR interpersonal distance OR social distance OR social withdrawal OR approaching OR peripersonal spatial OR physical proximity OR peripersonal Space OR peripersonal distance OR personal space regulation OR social approach OR personal space intrusion OR modulating interpersonal space) AND (fMRI OR neural OR neuronal OR fMRI investigation OR neural correlates OR neural activations OR numerical cognition OR different neural activations).

### 2.3. Quality Assessment of Each Study

This systematic review adopted a modified version of the Newcastle-Ottawa Quality Assessment Scale (NOS) (Supplementary Table S1). The NOS includes 3 categories of evaluation, with 9 items. The NOS tool is the classic evaluation tool for non-randomized controlled trial (non-RCT) studies and is recommended by the Cochrane Handbook. In brief, NOS rates study quality in terms of selection, comparability, and outcome. To specifically distinguish the level of evaluation of the NOS tool, each study was given a quality rating of “good”, “fair” or “poor”.

## 3. Results

**Figure 1:**
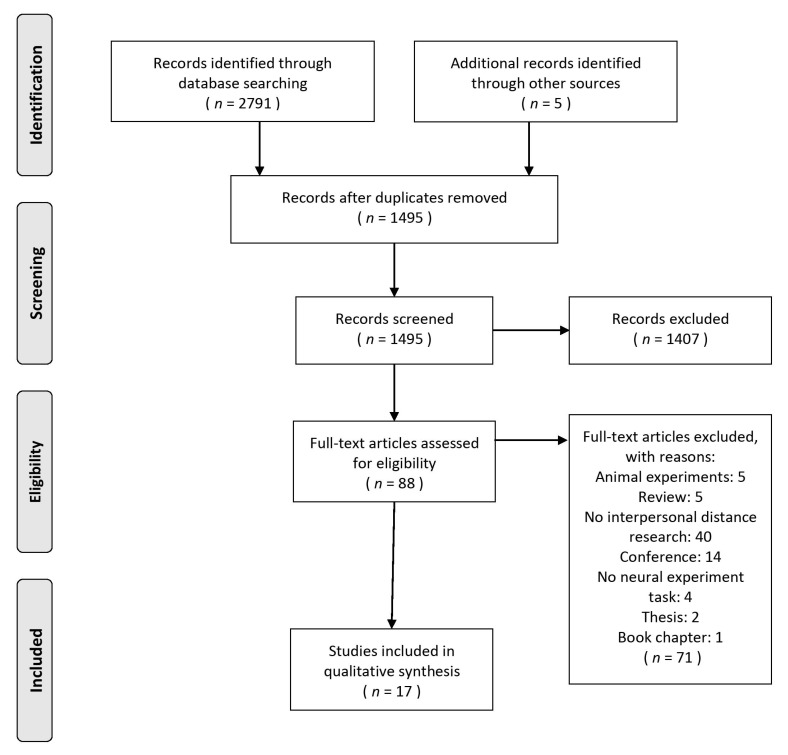
Flow diagram of the selection procedure.

17 articles that met the requirements were included in this review; they were selected from 2796 results. The flow diagram (Figure 1) is illustrated the study selection procedure.

### 3.1. Quality Assessment of Each Study

All articles included in the systematic review passed the quality assessment (Supplementary Table S1). Most of the included studies were of “good” quality rating. 12 of the articles received a score of 7–9 stars and were rated as “good” [40,41,42,44,45,46,47,48,49,50,51,52], while the other 5 articles received a score of 5–6 stars and were rated as “fair” [18,53,54,55,56].

### 3.2. Basic Information of the Studies

From Table 1, 11 studies were conducted with healthy individuals [18,40,41,45,47,48,49,50,52,54,56] and 6 were with participants who had psychiatric disorders [42,44,46,51,53,55]. 696 subjects (47.6% males, 52.4% females) took part in the IPD experiments while recording the neural activities. 595 were healthy subjects (46.6% males, 53.4% females) who reported no history of psychiatric or neurological illness, 101 were psychiatric subjects (53.5% males, 46.5% females) confirmed by professional assessment. 9 studies reported the handedness of subjects [18,40,44,45,47,49,50,53,56].

### 3.3. Study Design

The approaching and withdrawal task (9 studies) [41,42,44,46,47,48,50,52,54] and the modified computerized version of the comfortable interpersonal distance task (5 studies) [18,40,45,53,55] of the IPD-neural paradigms, were the dominant tasks that were adopted in the IPD neural tasks (Table 1). The IPD experiment tasks were computerized IPD paradigms [40,41] which were modified from classical IPD evaluation paradigms, like the good ecological stop-distance paradigm [57,58] and comfortable interpersonal distance (CID) [59]. Among them, the most popular computerized IPD paradigm that used in the fMRI studies was approaching and withdrawal stimuli. During this paradigm, subjects passively view stimuli (social or non-social/face or objects) moving forward to him or moving backward from him, to monitor the interpersonal motion process. In this paradigm, the neural data reflected the motion cognition features of subjects and the characteristics of social stimuli (face) cognition in a moving duration. Besides, the brain activities of a comfortable distance judgment were observed according to the CID task by EEG technique, where the subject imagines that he was the figure in the center of a circle and approached by other figures (strangers/friends) starting from the edge of the circle, and then presses the stop button at the distance where the subject feels the most comfortable. And other 3 types of the IPD-tasks evaluated the space between persons by indirect task score conversion, included the interpersonal space task [51], judgment task [56], and the choice task of social and non-social stimuli [49]. Overall, it is obvious that the results of the neural-IPD cognition were from a passive IPD pattern, while the processes of the IPD including active participation.

### 3.4. Neural Activity of IPD Processing

In this section, we showed findings from fMRI findings firstly, as the majority of studies have focused on the changes of brain regions. Then, we turn to present findings from the EEG during the IPD processing, followed by findings from the correlation of neural activation and IPD.

#### 3.4.1. Neural Activity of the fMRI Findings

As shown in Table 2, among the 12 IPD task reviews reported by the functional magnetic resonance imaging study, 11 reported changes in brain activities in the parietal lobe region of the subjects [41,42,44,45,46,47,48,50,51,52,54]. The increased brain activity was found in the dorsal intraparietal sulcus [41,42], inferior and superior parietal cortices [44,54], inferior and superior parietal lobules [47], these functional brain activations were observed in the computerized IPD tasks, during which subjects passively watched the stimuli approaching contrast stimuli withdrawal. IPD is a physical distance between two people. Spatial change and motion modulation occur during the processes of IPD interaction. The parietal lobe is well known for the cognition of space, especially self-space cognition [60]. Moreover, in patients with schizophrenia, self-spatial dissonance is associated with impairment of the parietal lobe [42]. In addition, the greater activation of the parietal lobe associated with the social information, such as the enhancement of DIPS to faces or human approach compares with cars or object approach [41,48], the increase in the inferior parietal lobule in the friends’ condition rather than stranger condition [45], the improvement of left inferior parietal region to approach male faces [46] and the right inferior parietal lobe increased in the condition of the approaching happy or angry faces compared to other emotion faces [47].

IPD is not only a motion modulation of the human being but also a complicated social behavior. 8 of 12 results of the included fMRI studies pointed out the activities in the frontal area of the brain during the IPD-tasks [41,42,45,46,47,49,50,52]. Firstly, the prefrontal cortex (PFC) associated with fear and anxiety were activated when processing social information. Greater left medial prefrontal cortex (mPFC) and ventromedial prefrontal cortex (vmPFC) changes were observed during the processing of approaching social stimuli compared with non-social stimuli [50]. In an IPD choice task with social and nonsocial stimuli, selection of social stimuli was accompanied by a significant increase in activity in the right medial frontal gyrus [49]. In a study of anxiety and social behavior in rats, it was found that activated basolateral amygdala and mPFC connection (BLA-mPFC) reduced social interactions in the resident-intruder test and stimulated social anxiety in rats [61]. In addition, mPFC plays an important role in the regulation of fear emotions generated by human cued fear conditions [62]. Furthermore, the vmPFC, which deals with negative emotions [63]. Second, Social relationships influence the adjustment of IPD, and we choose to stay a closer IPD with our friends. When interacting with friends, we are willing to allocate more attentional resources to them, we prefer to keep a closer IPD with familiar people. In a modified version of the CID task, the enhancement of left mPFC to friend IPD evaluation compared to stranger IPD evaluation was observed in the condition of oxytocin condition. While in the placebo condition, the changes of left mPFC decreased [45]. Thirdly, there is a gender effect in IPD, the close of female faces was associated with increased activity of OFC [46]. The OFC is the primary neural mechanism for emotion generation and is involved in complex decision-making.

Additionally, the changes of the motor cortex were found in 7 studies [41,42,44,45,46,48,50]. We found that the positive activation of the PMv [41,50] and the somatosensory cortex [44] were associated with stimulus approaching contrast stimulus withdrawal or static. The premotor cortex is responsible for managing the body’s motor control, which includes the processing of motor-sensory information and spatial guidance for reaching. IPD is a motion modulation processing, during which we observe the behavior of others. In particular, the impact of IPD changes occurs between others and us on our IPD adjustments. We also modulate the IPD with others to a distance that we feel at ease.

Furthermore, the emotional state has an impact on the neural changes of IPD cognition. 4 studies observed increased activation in the amygdala [44,46,47,54]. These neural activities were related to gender stimulus. There is a gender effect in IPD, for example, men tend to be far from men in IPD. In the present review, it was also found that the approach of male faces stimulated stronger amygdala activity than the approach of female faces. The amygdala is an emotional processing center that plays a key role in fear and reward effects [64]. It may indicate the proximity of a male provokes the amygdala response associated with fear.

#### 3.4.2. Functional Connectivity Response to IPD Tasks

Six out of the 12 fMRI studies tried to find the functional connectivity response to IPD tasks, while 5 studies had significant results associated with the IPD-tasks [41,42,49,50,51]. The study reported the significant connectivity with the DIPS and PMv seed regions (Approaching > Withdrawing face stimuli) [41]. Furthermore, the dorsal striatum which was selected as a seed, was found connectivity with the right and left occipital lobe, left thalamus, right parietal lobe, left superior frontal gyrus, and bilateral putamen in oxytocin or placebo condition during IPD task [49]. In addition, in approaching social stimuli, significant connections were found in the midbrain with bilateral premotor cortex and the midbrain with right dorsolateral prefrontal cortex compared to non-social stimuli [50]. Besides, compared to healthy controls, Autistic Spectrum Disorders (ASDs) showed the connectivity enhancement of the amygdala with DIPS and FFA (face fusiform gyrus), and the deceased connectivity of FFA to the DIPS and amygdala [51].

#### 3.4.3. ERPs in Response to IPD Tasks

According to Table 2, event-related potentials were examined in 5 studies [18,40,53,55,56], with IPD-task found to have an correlates on neural processing, including N1 [40,53,55], latency of N1 [55], P1 [40,53], alpha suppression [18], electrode O2 [18], lateralized N170 [56], and N2 posterior contralateral (N2pc) amplitudes [56]. During the IPD-tasks, the figure of the other person approaching the figure of subjects, the movements, and spatial stimuli the changes of N1 and P1 were verified to be neural markers during visuospatial processing. In research on visual space processing of N1, it was found that N1 not only deals with absolute spatial positions but also relates to the processing of relative spatial positions [65]. Furthermore, P1 and N1 amplitude were enhanced by spatial attention [66].

#### 3.4.4. Correlation between IPD and Neural Activity

Although changes in brain activity in the IPD neural task were discussed, does the founded brain activity reflect IPD in real contexts? In this literature review, the IPD-neural task showed medium significant correlations with the ecological IPD task, including positive and negative correlations.

Table 3 shows the results of the regression analyses of IPD and brain activity. On the one hand, greater N1 ERP amplitude was related to the increase in preferred distance [53]. On the other hand, both positive and negative results were found in the fMRI studies. IPD was positively associated with the increased activation of several brain areas. DIPS-PMv coupling was found positively associated with personal space permeability [41,42]. Compared with the increase in the DIPS [42], right amygdala [47], near-space network [48], right dorsal striatum (oxytocin condition) [49], and left FFA [51], the IPD enlarged. Furthermore, DIPS-PMv connectivity [42], right dorsal striatum (placebo condition) [49], connectivity strength between the midbrain and the left premotor cortex [50], connectivity from FFA to amygdala [51] had a negative relationship with IPD. It means the enhancement of the activation of these areas brings to the decrease of IPD.

Interestingly, the enhanced functional connectivity of the brain was accompanied by a decrease in IPD, implying the more active the functional connections, the shorter the IPD. It was reported that greater DIPS-PMv connectivity related to a smaller personal space size [41]. Enhanced DIPS-PMV connectivity means more motion modulation resources are consumed when processing closer and smaller IPD. Furthermore, connectivity strength between the midbrain and the left premotor cortex was found negatively correlated with IPD [50]. There was evidence that these areas were related to dealing with the peripersonal space during tool use and interpersonal motor interaction [67]. Previous researches pointed out that the larger the size of a social network, the greater the functional connectivity [68]. Overall, the increased functional connectivity may symbolize the intimacy of IPD between people and the engagement of social interaction.

IPD expands along with the activation of some brain activities. Greater activation of the right amygdala was found to have a relationship with the increase in preferred distances accompanying negative expressions [47]. The amygdala is associated with negative emotions, especially fear [69]. It may symbolize that the amygdala plays a role in IPD protection. When the emotion of external interaction was fear, sadness, or anger, the increased activity of the amygdala implies an avoidance mechanism of negative emotions and indicated the protection of self-space boundaries. Moreover, researchers pointed out that the parietal cortex was responsible for sensory and motor coordination, greater DIPS activation in the condition of approaching faces related to a larger IPD. Furthermore, A smaller N1, in the context of interpersonal distance, may be related to avoidance mechanisms. While in the case of ASD, an increase in N1 was related to the far IPD [53], which indicated the dysfunction of ASD.

To sum up the above, findings in the neural-IPD generally indicate that the parietal lobe, prefrontal area, motor cortex, and amygdala may play an important role during the IPD processing. Although there was some exploration of the found neural areas, little is known specifically about the connectivity of these regions. Besides, the neural activities of computerized-IPD have a moderate correlation with IPD behavior. Hence, it will be significant to investigate the neural connectivity of the brain area. And, Neural IPD experimental paradigms with better ecological validity and reproduction of real IPD interaction processes will be critical to deeply understand IPD brain activity.

## 4. Discussion

IPD is a hot topic in the fields of sociology and psychology, but it has been combined with neuroscience in the past ten years. The earliest literature we included was a study published in 2013. Although the similarity concepts like peripersonal space and near-body space were discussed by researchers, peripersonal space links to the space around the body, with which “near” or arm’s length of the body, while the space occupied by the body is called the near-body space [70]. Studies on these body spaces had revealed the associated neural coding of body schemas [71]. However, these spaces are related to movement, control, and tool use and are different from the spatial concept of IPD explored in this review. In the past, cognitive neuroscience has not yet explored IPD. It may be that the mainstream of neuroscience researches focused on internal cognitive processes, such as perceptual processing [72], thinking [73], and memory [74]. Neuroscience research techniques, such as EEG and fMRI, have operating limitations for the IPD experimental process, so there is a requirement for technological breakthroughs for the exploration of IPD, which is an external behavior. With the development of cognitive neuroscience, researchers began to interest in external behaviors, computerized IPD tasks had appeared, and offered the possibilities to explore the neural basis of IPD.

This systematic review examined the IPD processing neural basis. Overall, the passive experiment paradigm is the main methodology of neural IPD exploration. And, The findings from the current review pointed out the parietal and motor lobe may be mainly related to IPD regulation processes, while the prefrontal lobe may be responsible for IPD changes with different social relationships, such as between acquaintances and strangers, in addition, the amygdala was thought to be associated with emotion-related IPD changes. Furthermore, evidence from fMRI and EEG indicates a medium effect between neural changes and IPD behavior. 

It is vital to further discuss the findings. Above all, it is very clear that neural changes in IPD cognition are an integrated and complex process that involves not only spatial cognition and motor adjustment but also is influenced by social factors and emotions. Substantial evidence of the complexity of neural activity in the cognitive processes of IPD. Multiple EEG components and amplitudes, like N1 and P1 changes during IPD, and evidence from fMRI suggests that these diverse neural changes are complicated, and both the region of interest (ROI) analysis and the whole brain analysis reveal multiple brain regions that were activated and connected during the IPD processing.

Additionally, current studies on the analysis of functional connectivity between brain regions involved in IPD processes are insufficient. Only a small number of studies have addressed the exploration of functional connectivity associated with IPD stimulation. Furthermore, the focus of these studies has primarily been on understanding functional connectivity between the DIPS and PMv, dorsal striatum as a seed connected with occipital lobe and other areas, midbrain periaqueductal grey (PAG) as a seed correlated with premotor cortex and dorsolateral prefrontal cortex, and the connectivity between AMY (amygdala)-DIPS, and AMY-FFA. These results suggested the high involvement of the DIPS region in functional connectivity in response to IPD stimuli, though more research is required. The neural activity of the IPD process does not work in isolation, so it is necessary to build systematic neural networks to understand the neural functions that collaborate.

Furthermore, there appears to be a passive model of IPD processing which has been a longstanding paradigm in IPD fMRI studies. The passive experimental paradigm includes a large number of looming faces tasks and a few IPD-tasks, such as computerized CID tasks and interpersonal space tasks. Although these experimental tasks are passive IPD paradigms, computerized-CID has processes of IPD assessment and selection of acquaintances and strangers, whereas looming faces tasks are completely passive in the presentation of receptive stimuli. Thus, relatively active PFC activity was observed in the computerized-CID task, whereas brain areas in the looming faces tasks presented more movement-related brain activity changes. However, these types of IPD-tasks cannot fully reveal the neural processing of IPD. In essence, IPD is a reciprocal social behavior in which we both actively approach others and are approached by others. In the behavioral study of IPD based on Virtual Reality (VR) technology, we can find the experimental paradigms of active approach and passive approach [15,75,76]. During the experiments reviewed so far, participants were passively approached by the character stimuli, without actively approaching others for exploring brain activity. From a passive perspective, the majority of neural-IPD studies investigating the activity and connectivity of IPD processing. While it is well established that the parietal lobe plays a role in IPD processing, a passive model ignores the relative contributions of active participation of the IPD, particularly the executive cognitive function and decision-making (i.e., PFC), and other important structures related to personal relationships understanding, such as the anterior cingulate cortex (ACC) which is located in the limbic system.

Heterogeneity between studies is influenced by a variety of factors, which makes the results inconsistent between studies and the work of comparing studies difficult. First, the functional brain techniques of EEG and fMRI have different focuses and advantages, and the analysis of data from EEG or fMRI, with inconsistent ROIs between studies, yields inconsistent results. For example, one EEG focuses on observing N1 [40], while another examines N170 [56]. Second, inconsistent data analysis methods make it difficult to compare results. For example, to explore the relationship between IPD neural activity and IPD behavior, some studies use Pearson correlation analysis [53], while some fMRI studies use Dynamic Causal Modeling (DCM) [51] or Voxelwise regression analyses [42]. Third, studies have inconsistent control groups or baselines. For example, although the studies were conducted with healthy individuals, the studies focused differently, leading to differences in the control group. A study included healthy individuals with high social anxiety (HSS) as subjects and healthy individuals with low social anxiety as controls [41], to discuss the IPD neural characteristics of HSS. While the research focused on the effect of oxytocin on IPD cognition, it grouped the participants into the Oxytocin group (subjects) and placebo group (controls) [49]. Therefore, different control groups may lead to different neural activity outcomes, and study results become difficult to compare. All of these points can be factors that contribute to the difficulty of comparison between studies.

Previous studies have explored the neural activity mechanisms of IPD in interpersonal states, however, the following gaps exist. IPD is a complex and integrated process that includes the adjustment of body space and is also influenced by a combination of social factors, as well as the active participation of the individual. Therefore, the experimental paradigms in this systematic review didn’t present the process of IPD completely and systematically. Although passive IPD research provides a way to understand social behavior, a paradigm closely related to personal defenses and private space, active IPD participation is key to building interpersonal distance. Therefore, we suggest that a standard IPD-neural experimental paradigm requires both active and passive interpersonal distance interaction processes. In addition, IPD interactions include many cognitive and behavioral processes, such as active thinking, evaluation, and adjustment of the IPD of individuals. Future experimental designs can consider the above aspects and try to measure the thinking process, action process, and adjustment process of IPD separately during the interpersonal interaction experiments, providing discussion and exploration of neural mechanisms in different IPD interaction stages. Furthermore, the observed correlation between brain activity and IPD behavior was a moderate level, while some studies reported weak or no correlation between them. Computerized IPD tasks may not effectively respond to real IPD processing and may not be a valid reflection of real IPD processing. Therefore, flexible, convenient, and interference-resistant brain activity acquisition devices, such as functional near-infrared spectroscopy (fNIRS), are needed for future studies. fNIRS is a brain activity recording tool developed in the last two decades, with good resistance to interference and good spatial and temporal separation rates. fNIRS is particularly suitable for experiments in natural contexts, and this feature of fNIRS provides the possibility of active IPD-evaluation, where subjects can wear NIRS devices on their heads and take real action to assess IPD with others [77]. In addition, the NIRS device is particularly suitable for multi-person tasks, offering the possibility to explore inter-brain synchronization for multi-person IPD interactions. Besides, IPD is a diverse process of human interaction and multiple experimental paradigms, like experimental paradigms combined with VR technology [78] or interactive IPD experimental tasks, such as the one in which both individuals feel the most comfortable with each other.

## 5. Limitation and Future Research

Although this review has initially sorted out the neural mechanisms of IPD, it still has limitations. The current review did not select a specific population as the subject of the study, and the results included both healthy people and people with mental disorders or traits. Moreover, the studies included in this review used different IPD experimental paradigms and considered different emphases. Besides, most of the studies had small sample sizes and no sample size estimation was performed before the experiments were implemented. Therefore, this will provide important information for future studies on this topic that need to consider the experimental paradigm and how to compare the differences between groups.

## 6. Conclusions

This systematic review explored the neural changes during the IPD task and the correlation with physical interpersonal distance. This review provides the neural activity of the IPD interaction process. Additionally, factors affecting real IPD interaction that act on the neuroactive processes of IPD and show consistent results. However, due to the complicated nature of IPD, the ecological validity of the neural-IPD tasks was insufficient, and the tasks require further attention towards the activity of human beings during IPD interaction. Therefore, the neural exploration of IPD is looking forward to multiple IPD research topics and high ecological and valid neural-IPD paradigms.

## Figures and Tables

**Table 1:** Basic information and study design for the included studies.

Author (et al.) (Year)	Participants	Age (M ± SD)	Gender (Male)	Handedness (R)	IPD Neural Task	Experiment Design	Image Technical
Anat Perry (et al.) (2013) [40]	48 undergraduates low social anxiety (SA):22(12 female) high SA:22(10 female) (4 subjects were excluded from data analysis)	27.5 ± 2.9 23–39	48 (24)	40	A modified computerized version of the comfortable interpersonal distance (CID)	2 Figures (close friend/stranger) × 8 Radii (0°/45°/90°/135°/180°/225°, 270°/315°)	Event-related potential (ERP)
Daphne J. Holt (et al.) (2014) [41]	8 healthy subjects 14 healthy subjects (later enrolled)	26.4 ± 4.7 24.6 ± 4.5	8 (4) 14 (8)	-	Approach and Withdrawal stimuli	2 Motions (approach/withdrawal) × 3 Stimuli (faces/cars/spheres)	Functional magnetic resonance imaging (fMRI)
Daphne J. Holt (et al.) (2015) [42]	15 schizophrenia (SCZ) 14 healthy control (CON)	30.1 ± 9.1 26.0 ± 6.5	-	-	Approach and Withdrawal stimuli	2 Motions (approach/withdrawal) × 2 Stimuli (faces/cars)	fMRI
Anat Perry (et al.) (2015) [53]	13 typical participants 13 participants with Autistic Spectrum Disorder (ASD)	24 ± 0.46 25 ± 1.24	13 (13) 13 (12)	13 (12) 13 (13)	A modified version of the CID task	2 Figures (Stranger/Friend) × 8 Entrances (0°/45°/90°/135°/180°/225°, 270°/315°)	ERP
Anne Schienle (et al.) (2015) [44]	25 Borderline Personality Disorder (BPD) patients 25 healthy controls	26.9 ±7.8 27.2 ±7.6	25 (0) 25 (0)	20 20	Still and approaching of facial expressions stimuli	2 Motions (still/approaching) × 3 Facial expressions (angry/disgusted/neutral)	fMRI
Anat Perry (et al.) (2016) [18]	42 undergraduate	22.53 ± 4.29	42 (0)	42 (41)	A modified version of the CID task	3 Figures (male friend/male stranger/screen) × 4 Entrances (0°/90°/180°/270°)	Electroencephalograph (EEG)
Albert Wabnegger, Verena Leutgeb and Anne Schienle (2016) [54]	30 healthy participants	27.3± 8.1	30 (0)	-	Pictures of neutral facial expressions stimuli	2 Motions (static/approaching) × 2 Faces (male/female)	fMRI
Daniela Cohen (et al.) (2017) [45]	19 healthy participants	26.05 ± 3.51	19 (19)	19	A modified version of the CID task	2 Figures (friend/stranger) × 4 Entrances (0°/90°/180°/270°)	fMRI
Anne Schienle (et al.) (2017) [46]	17 violent offenders18 nondelinquent controls	34.82 ± 12.54 37.89 ± 9.21	17 (17) 18 (18)	-	Pictures of neutral facial expressions stimuli	2 Motions (Static/approaching) × 2 Facial gender (man/women)	fMRI
Joana B. Vieira (et al.) (2017) [47]	23 healthy participants	20.96 ± 2.48	23 (11)	23	Approaching and withdrawal facial expressions stimuli	2 Motions (approaching/withdrawal) × 5 Facial expressions (anger, fear, happiness, sadness, neutral)	fMRI
Eti Ben Simon, Matthew P. Walker (2018) [48]	80 healthy adults	20.2 ± 1.5	80 (71)	-	Social distance task—computerized	2 Video stimuli (human/objects)	fMRI
Daniela Cohen (et al.) (2018) [49]	24 healthy participants	28.02 ± 2.69	24 (24)	24	Choice task of social and non-social stimuli	2 Stimuli (social/non-social) × 3 Sizes (small/medium/larger distance)	fMRI
Orly Rubinsten (et al.) (2020) [55]	11 developmental dyscalculia (DD) 12 typically developing controls (TD)	29 ± 2 30 ± 4	11 (11) 12 (7)	-	A modified computerized version of the CID	4 Figures (close friend/stranger/ball/artificial figure presented as a “griple”) × 8 Entrances (0°/45°/90°/135°/180°/225°, 270°/315°)	ERP
Joana B. Vieira, Stephen R. Pierzchajlo & Derek G.V. Mitchell (2020) [50]	30 healthy volunteers	21.90 ± 3.51	30 (6)	30	Experimental tasks: Social and non-social stimuli of varying threat levels	2 Pictures (face/insects) × 2 Threats (high/low) × 2 Motions (approaching/withdrawal)	fMRI
Aimee Martin, Stefanie I. Becker and Alan J. Pegna (2021) [56]	Experiment1: 15 healthy subjects Experiment2: 30 healthy subjects	22.73 ± 2.02 24.43 ± 9.66	15 (5) 30 (14)	15 28	Experiment 1: The task of judging letter changes with dynamic emotional faces Experiment 2: Gender judgment task	Experiment 1: 2 Face orientations (Upright, Inverted) × 2 Looming expression (Fearful, Neutral) × 2 Laterality of looming stimuli (Contralateral, Ipsilateral) Experiment 2: 2 Face expressions (Fearful, Neutral) × 4 Face gender combinations (male-male, female-female, male-female and female-male) × 2 spaces (50 cm near space,120 cm far space)	ERP
Claudia Massaccesi (et al.) (2021) [51]	20 high-functioning ASD adults 20 controls	34.25 ± 11.65 33.05 ± 12.33	20 (14) 20 (14)	-	Interpersonal Space task (Step 1–5 forward videos)	5 Steps (step 1/step 2/step 3/step 4/step 5)	fMRI
Nasiriavanaki, Z. (et al.) (2021) [52]	130 healthy subjects - a Reference Sample (80) - a Test Sample (50)	19.45 ± 1.4 19.6 ± 1.4	80 (25) 50 (14)	-	Approach and Withdrawal stimuli	2 Stimuli (human faces/cars) × 2 Gender (female face/male face) × 2 Motions (approaching/withdrawal)	fMRI

-: no report; CID: comfortable interpersonal distance; SA: social anxiety; SCZ: schizophrenia; CON: healthy control; ASD: Autistic Spectrum Disorder; BPD: Borderline Personality Disorder; DD: developmental dyscalculia; TD: typically developing controls; dmPFC: dorsomedial prefrontal cortex; ERP: Event-related potential; fMRI: Functional magnetic resonance imaging; EEG: Electroencepha-lograph.

**Table 2:** Neural activation and functional-connection findings for the included studies.

Author (et al.) (Year)	Participants	Age (M ± SD)	Gender (Male)	Handedness (R)	Data Analysis	Neural Findings	
						Neural Activation	Functional Connectivity
Anat Perry (et al.) (2013) [40]	48 undergraduates low SA:22 (12 female) high SA:22 (10 female) (4 subjects were excluded from data analysis)	27.5 ± 2.9 23–39	48 (24)	48 undergraduates low SA:22 (12 female) high SA:22 (10 female) (4 subjects were excluded from data analysis)	ERPs: N1, P1 Late Positive Potential (LLP)	① High SA group > Low SA group ↓P1 ↓N1 ② Stranger condition > Friend condition ↑N1 ③ R Hemisphere > L Hemisphere (low SA group only) ↑N1	-
Daphne J. Holt (et al.) (2014) [41]	8 healthy subjects 14 healthy subjects (later enrolled)	26.4 ± 4.7 24.6 ± 4.5	8 (4) 14 (8)	8 healthy subjects 14 healthy subjects (later enrolled)	Region of interest (ROI): dorsal intraparietal sulcus (DIPS), ventral premotor cortex, precuneus (PMv) Whole-brain analysis	① Approach faces > Withdrawal faces ↑DIPS, PMv, mid-cingulate gyrus, dorsal precentral, middle frontal gyri, middle occipital and inferior temporal gyri, and ventral superior parietal gyrus ② Faces > cars, Approach > Withdrawal ↑DIPS and PMv	Significant connectivity with the DIPS and PMv seed regions (Approaching > Withdrawing face stimuli) (n = 17)
Daphne J. Holt (et al.) (2015) [42]	15 schizophrenia (SCZ) 14 healthy control (CON)	30.1 ± 9.1 26.0 ± 6.5	-	15 schizophrenia (SCZ) 14 healthy control (CON)	ROI: DIPS, PMv Whole-brain analysis	① Controls and schizophrenic patients: approaching faces > withdrawing faces: ↑DIPS and PMv ② Schizophrenic patients > Controls: ↑left DIPS, ↑left lateral frontal cortex,↑right middle temporal gyrus	DIPS-PMv connectivity (CON and SCZ) DIPS (Approaching > Withdrawing Faces)
Anat Perry (et al.) (2015) [53]	13 typical participants 13 participants with ASD	24 ± 0.46 25 ± 1.24	13 (13) 13 (12)	13 typical participants 13 participants with ASD	ERP Analysis: N1, P1	① R hemisphere > L hemisphere ↑P1 (within all subjects) ② Stranger > friend ↑N1	-
Anne Schienle (et al.) (2015) [44]	25 BPD patients 25 healthy controls	26.9 ± 7.8 27.2 ± 7.6	25 (0) 25 (0)	25 BPD patients 25 healthy controls	ROI: amygdala, the insula, the premotor cortex, the putamen, and parietal regions Whole-brain voxel intensity tests	Approaching > Still:↑right amygdala, ↑several parietal regions (primary somatosensory cortex, inferior parietal region, intraparietal sulcus)	-
Anat Perry (et al.) (2016) [18]	42 undergraduate	22.53 ± 4.29	42 (0)	42 undergraduate	Electroencephalograph (EEG) analysis: Alpha suppression, Hemispheres	① High sensory sensitivity group > Low sensory sensitivity group ↑Alpha suppression,↑electrode O2 ② High sensory sensitivity group > low sensory sensitivity group, occipital sites > central sites > frontal sites ↑ Alpha suppression ③ Friend > computer screen ↑ Alpha suppression ④ Far distance > middle distance > near distance ↑Alpha suppression (occipital and central sites)	-
Albert Wabnegger, Verena Leutgeb and Anne Schienle (2016) [54]	30 healthy participants	27.3 ± 8.1	30 (0)	30 healthy participants	ROI: amygdala, putamen, and parietal regions Whole-brain voxel intensity tests	① Approaching stimuli > static stimuli:↑bilateral inferior, ↑superior parietal cortices,↑intraparietal sulci,↑left primary somatosensory cortex (SI), ↑occipital areas ② Approaching male stimuli > Approaching female stimuli: ↑R amygdala	-
Daniela Cohen (et al.) (2017) [45]	19 healthy participants	26.05 ± 3.51	19 (19)	19 healthy participants	Whole-brain analysis	① Friend > stranger: ↑R temporal lobe/occipital lobe/middle temporal gyrus,↑ L superior frontal gyrus/premotor/subthalamic nucleus/cingulate gyrus/inferior frontal gyrus/parahippocampal gyrus/middle occipital gyrus/inferior parietal lobule ② Oxytocin friend > Oxytocin stranger: ↑L medial prefrontal cortex, ↑R anterior cingulate, ↑R posterior-anterior cingulate, ↓R parahippocampal gyrus ③ Placebo friend > Placebo stranger: ↓L medial prefrontal cortex, ↓R anterior cingulate, ↓R posterior-anterior cingulate, ↑R parahippocampal gyrus	-
Anne Schienle (et al.) (2017) [46]	17 violent offenders 18 nondelinquent controls	34.82 ± 12.54 37.89 ± 9.21	17 (17) 18 (18)	17 violent offenders 18 nondelinquent controls	ROI: amygdala, the insula, the premotor cortex, and parietal regions Whole brain voxel intensity tests	① controls > offenders:↑inferior parietal ② Approaching > Static: ↑fronto-parietal regions (premotor cortex, SI, dorsolateral prefrontal cortex (DLPFC), superior/inferior parietal region),↑insula activation ③ Female faces > male faces: ↑Orbitofrontal cortex(OFC)activation ④ Offenders > Controls: Approaching > Static: ↑ insula activation ⑤ Offender > Controls, Male > Female: Approaching > Static:↑R insula activation ⑥ Male > Female, Approaching > Static: ↑L amygdala,↑L inferior parietal region	-
Joana B. Vieira (et al.) (2017) [47]	23 healthy participants	20.96 ± 2.48	23 (11)	23 healthy participants	ROI: amygdala Whole-brain analysis	① Approaching > Withdrawal: ↑bilateral visual cortex,↑fusiform gyrus, ↑R inferior parietal lobule (IPL),↑superior parietal lobules (SPL),↑R amygdala, ↑bilateral anterior insula (AI), DLPFC ② Happiness, Angry > fear, sadness, neutral:↑L dorsomedial prefrontal cortex (dmPFC),↑R OFC, R Inferior frontal gyrus (IFG),↑R Inferior parietal lobule (IPL) ③ Approaching > Withdrawal, Happiness, Angry > fear, sadness, neutral: ↑insula (bilaterally),↑L IFG ④ Approaching > Withdrawal, Sadness > happiness, angry, fear, neutral:↓insula (bilaterally) ⑤ Withdrawal > Approaching, sadness > happiness, angry, fear, neutral ↑ventrolateral prefrontal cortex (vlPFC) (bilaterally) ⑥ Approaching > Withdrawing, Happiness > sadness, angry, fear, neutral ↑vlPFC (bilaterally)	-
Eti Ben Simon, Matthew P. Walker (2018) [48]	80 healthy adults	20.2 ± 1.5	80 (71)	80 healthy adults	ROI: Near Space network Theory of mind (ToM) network	① Human approach > object approach, sleep-deprivation > Sleep rested:↑Near Space network (dorsal intraparietal sulcus and ventral premotor cortex) ② Human approach > object approach, Sleep rested > sleep-deprivation:↑Theory-of-Mind network (temporal–parietal junction and precuneus)	-
Daniela Cohen (et al.) (2018) [49]	24 healthy participants	28.02 ± 2.69	24 (24)	24 healthy participants	ROI: Right dorsal striatum, dmPFC whole-brain analysis	① social stimulus > non-social stimulus:↑R medial frontal gyrus ② Oxytocin > Placebo:↓L anterior cingulate (ACC),↓R culmen ③ Oxytocin > Placebo, social stimulus > non-social stimulus:↑R dorsal striatum	A connectivity Psychophysiological interaction (PPI) analysis dorsal striatum as a seed: Placebo condition: R Occipital lobe, L thalamus, R parietal lobe, L superior frontal gyrus, L occipital lobe Oxytocin condition: R occipital lobe, R putamen, L occipital lobe, L putamen
Orly Rubinsten (et al.) (2020) [55]	11 developmental dyscalculia (DD) 12 typically developing controls (TD)	29 ± 2 30 ± 4	11 (11) 12 (7)	11 developmental dyscalculia (DD) 12 typically developing controls	ERP analysis: N1	DD > TD:↑Latencies N1,↑N1	-
Joana B. Vieira, Stephen R. Pierzchajlo & Derek G.V. Mitchell (2020) [50]	30 healthy volunteers	21.90 ± 3.51	30 (6)	30 healthy volunteers	Whole-brain analysis	① Social stimuli > Non-social stimuli (approach/withdrawal event): ↑R face fusiform area (FFA),↑bilateral temporoparietal junction (TPJ), and↑L medial prefrontal cortex (MPFC) ② First static image of each trail social stimuli > non-social stimuli:↑R TPJ, ↑bilateral FFA, ↑Ventromedial prefrontal cortex (vmPFC) ③ Approach > Withdrawal:↑R midbrain periaqueductal gray (PAG),↑R insula,↑R PMv extending to the dorsolateral prefrontal cortex,↑bilateral superior parietal lobule ④ Near > Far:↑midbrain (PAG)	Approach Social stimuli > Approach Non-social stimuli A midbrain PAG seed: bilateral premotor cortex and R dorsolateral prefrontal cortex
Aimee Martin, Stefanie I. Becker and Alan J. Pegna (2021) [56]	Experiment1: 15 healthy subjects Experiment2: 30 healthy subjects	22.73 ± 2.02 24.43 ± 9.66	15 (5) 30 (14)	Experiment1: 15 healthy subjects Experiment2: 30 healthy subjects	ERPs analysis: N170, N2 posterior contralateral (N2pc)	Experiment 1: ① Looming fearful upright face contralateral amplitudes > ipsilateral amplitudes:↓l-N170,↓N2pc ② Upright looming fearful face > inverted neutral looming face: ↓l-N170 ③ Upright looming fearful face > inverted looming fearful face:↓N2pc Experiment 2: ④ Close faces > far faces:↑l-N170 ⑤ Contralateral amplitudes > ipsilateral amplitudes (fearful face):↓l-N170,↓N2pc (close distance) ⑥ fearful faces > neutral face (close/far distance):↓l-N170	-
Claudia Massaccesi (et al.) (2021) [51]	20 high-functioning ASD adults 20 controls	34.25 ± 11.65 33.05 ± 12.33	20 (14) 20 (14)	20 high-functioning ASD adults 20 controls	Task-based univariate fMRI analysis	CTR > ASD: bilateral dIPS, R human middle temporal visual area (hMT+/V5),L Fusiform Gyrus (FFA)	ASDs > CTRs:↑AMY- dIPS, AMY-FFA,↓FFA- dIPS,↑dIPS – AMY, ↓FFA -AMY
Nasiriavanaki, Z. (et al.) (2021) [52]	130 healthy subjects - a Reference Sample (80) - a Test Sample (50)	19.45 ± 1.4 19.6 ± 1.4	80 (25) 50 (14)	130 healthy subjects - a Reference Sample (80) - a Test Sample (50)	ROIs: peripersonal space (PPS) network Whole-brain analysis	① Peripersonal space (PPS)network responses (Face Approach > Withdrawal) ↑R and L superior frontal cortex (SFC), ↑R and L medial parietal cortex (MPC), ↑R and L superior parietal cortex (SPC) ② Inside > outside the personal space boundary:↑PPS network ③ Faces approach > cars approach: ↑PPS network	No significant correlations were found

↑: enhance, increase; ↓: decline, decrease; >: contrast; ROI: region of interest; R: right; L: left; LLP: late positive potential; SA: social anxiety; ERPs: event-related po-tentials; BPD: Borderline Personality Disorder; EEG: Electroencephalograph; SI: primary somatosensory cortex; DIPS: dorsal intraparietal sulcus; PMv: ventral premotor cortex; CON/CTR: controls; SCZ: schizophrenia; DLPFC: dorsolateral prefrontal cortex; dmPFC: dorsomedial prefrontal cortex; OFC: orbitofrontal cortex; IPL: inferior parietal lobule; SPL: superior parietal lobules; AI: anterior insula; dmPFC: dorsomedial prefrontal cortex; IFG: inferior frontal gyrus; IPL: inferior parietal lobule; vlPFC: ventrolateral prefrontal cortex; ToM: Theory of mind; ACC: anterior cingulate; PPI: Psychophysiological interaction; DD: developmental dyscalculia; TD: typically developing controls; ASD: autistic spectrum disorder; FFA: face fusiform area; TPJ: temporoparietal junction; MPFC: medial prefrontal cortex; PAG: midbrain periaqueductal grey; N2pc: N2 posterior con-tralateral; AMY: amygdala; PPI: psychophysiological interaction; SFC: superior frontal cortex; MPC: medial parietal cortex; SPC: superior parietal cortex; PPS: peripersonal space.

**Table 3:** IPD outcomes and their relationship with neural outcomes for the included studies.

Author (et al.) (Year)	Participants	Age (M ± SD)	Gender (Male)	Handedness (R)	IPD Task	IPD Index (Correlation)	Details of the Relationship between IPD and Neural Outcomes	r/z
Daphne J. Holt (et al.) (2014) [41]	8 healthy subjects 14 healthy subjects (later enrolled)	26.4 ± 4.7 24.6 ± 4.5	8 (4) 14 (8)	-	Stop-Distance paradigm	personal space size (pps) (−) personal space permeability (psp) (+)	①↑DIPS-PMv(dorsal intraparietal sulcus-ventral premotor cortex) functional coupling,↓personal space size ②↑DIPS-PMv functional coupling,↑ personal space permeability	Pearson correlation: pps r = −0.55 * Voxelwise regression analyses: pps z1 = 3.4 ** (−) psp z2 = 3.6 ** (+)
Daphne J. Holt (et al.) (2015) [42]	15 schizophrenia (SCZ) 14 healthy control (CON)	30.1 ± 9.1 26.0 ± 6.5	-	-	Stop-Distance paradigm	personal space size (+/−) personal space permeability	①↑DIPS activation (approaching > withdrawing faces), ↑personal space size ②↑DIPS–PMv connectivity,↓personal space size	Pearson correlation: r (left DIPS) = 0.62 * (CON) r (right DIPS) = 0.56 * (SCZ) Voxelwise regression analyses: pps z (CON) = 3.53 ** (−) pps z (SCZ) = 3.47 ** (−)
Anat Perry (et al.) (2015) [53]	13 typical participants 13 participants with ASD	24 ± 0.46 25 ± 1.24	13 (13) 13 (12)	13 (12) 13 (13)	Stop distance paradigm	Average preferred distances (+)	ASD group: ↑N1 ERP amplitude, ↑Average preferred distances	r = 0.62 *
Daniela Cohen (et al.) (2017) [45]	19 healthy participants	26.05 ± 3.51	19 (19)	19	A modified version of the comfortable interpersonal distance (CID) task	Distance index score -friend -stranger (+) -Oxytocin—Placebo (friend) -Oxytocin—Placebo (stranger) (−)	①↓dmPFC,↓distance index score (stranger) ②↓dmPFC,↑Oxytocin - Placebo distance index score (stranger)	Not report
Joana B. Vieira (et al.) (2017) [47]	23 healthy participants	20.96 ± 2.48	23 (11)	23	Computerized Interpersonal Distance Task	Computerized desired distance -sadness (+) -happiness -fear (+) -angry (+) -neutral	↑R amygdala ↑distance to angry,↑distance to sad,↑distance to fearful	r (angry) = 0.61 ** r (sad) = 0.527 ** r (fearful) = 0.504 *
Eti Ben Simon, Matthew P. Walker (2018) [48]	80 healthy adults	20.2 ± 1.5	80 (71)	-	Social distance task—computerized	sleep deprivation (SD) distance - sleep rested (SR) distance (+)	↑Near Space network (human > object approach),↑social distance (Sleep deprivation-Sleep rest)	r = 0.53 *
Daniela Cohen (et al.) (2018) [49]	24 healthy participants	28.02 ± 2.69	24 (24)	24	Choice task of social and non-social stimuli	Distance behavioral scores Placebo condition (−) Oxytocin condition (+)	↑Right dorsal striatum -↓distance scores (Placebo condition) -↑distance scores (Oxytocin condition)	r = −0.1 (Placebo) r = 0.3 (Oxytocin)
Joana B. Vieira, Stephen R. Pierzchajlo & Derek G.V. Mitchell (2020) [50]	30 healthy volunteers	21.90 ± 3.51	30 (6)	30	Computerized distance task “Stop-distance” task	percentage of stimulus (−) physical distance (−)	↑connectivity strength between the midbrain and the left premotor cortex ↓percentage of stimulus ↓physical distance	r1 = −0.409 * r2 = −0.374 *
Claudia Massaccesi (et al.) (2021) [51]	20 high-functioning ASD adults 20 controls	34.25 ± 11.65 33.05 ± 12.33	20 (14) 20 (14)	-	Interpersonal Space Task	Averaged comfort ratings(+/−)	↑L fusiform face area (FFA),↑Averaged comfort rating (all participants) ↓comfort,↑connectivity from FFA to the amygdala	r = 0.455 * connection parameters: 0.27

+: positive; −: negative; ↑: Enhance, increase; ↓: decline, decrease; * *p* < 0.05; ** *p* < 0.01.

## Data Availability

Not applicable.

## Supplementary Materials

The following are available online at https://www.mdpi.com/article/10.3390/brainsci11081015/s1, Table S1: Adapted version of the NEWCASTLE—OTTAWA QUALITY ASSESSMENT SCALE.
