# Supplementary material for: Neural Alterations in Interpersonal Distance (IPD) Cognition and Its Correlation with IPD Behavior: A Systematic Review
Source: Brain Sci. 2021 Jul 30;11(8):1015. doi: 10.3390/brainsci11081015 (PMC8394299; doi:10.3390/brainsci11081015)
Supplement: Supplementary file 1 [file brainsci-11-01015-s001.zip › brainsci-1274916-supplementary.pdf]

Supplementary 1: Quality Assessment Checklist

Table S1: Adapted version of the NEWCASTLE - OTTAWA QUALITY ASSESSMENT SCALE.

Authors	Year	Selection				Comparability	Outcome			Quality
		Representativeness of the study population	Same or similar selection of the subjects	Ascertainment of the exposure	Demonstration that outcome of interest was not present at start of study	Comparability of studies on the basis of the design or analysis	Assessment of outcome	In-depth and comprehensive data analysis（Functional connectivity/whole brain analysis）	Exploring the correlation between IPD brain activity and behavior	
Anat Perry (et al.)	2013	*	*	*	*	**	*			Good
Daphne J. Holt(et al.)	2014	*	*		*	**	*	*	*	Good
Daphne J. Holt(et al.)	2015	*		*	*	**	*	*	*	Good
Anat Perry(et al.)	2015	*		*	*	*	*		*	Fair
Anne Schienle (et al.)	2015	*		*	*	**	*	*		Good
Anat Perry(et al.)	2016	*	*	*	*	*	*			Fair
Albert Wabnegger,	2016	*		*	*	*	*	*		Fair

Authors	Year	Selection				Comparability	Outcome			Quality
		Representativeness of the study population	Same or similar selection of the subjects	Ascertainment of the exposure	Demonstration that outcome of interest was not present at start of study	Comparability of studies on the basis of the design or analysis	Assessment of outcome	In-depth and comprehensive data analysis（Functional connectivity/whole brain analysis）	Exploring the correlation between IPD brain activity and behavior	
Verena Leutgeb and Anne Schienle										
Daniela Cohen(et al.)	2017	*	*	*	*	*	*	*	*	Good
Anne Schienle(et al.)	2017	*		*	*	**	*	*		Good
Joana B. Vieira(et al.)	2017	*	*	*	*	**	*	*	*	Good
Eti Ben Simon,Matthew P. Walker	2018	*	*	*	*	**	*		*	Good
Daniela Cohen(et al.)	2018	*	*	*	*	*	*	*	*	Good
Orly Rubinsten (et al.)	2020	*		*	*	**	*			Fair
Joana B. Vieira, Stephen R. Pierzchajlo & Derek G.V. Mitchell	2020	*	*		*	**	*	*	*	Good

Authors	Year	Selection				Comparability	Outcome			Quality
		Representativeness of the study population	Same or similar selection of the subjects	Ascertainment of the exposure	Demonstration that outcome of interest was not present at start of study	Comparability of studies on the basis of the design or analysis	Assessment of outcome	In-depth and comprehensive data analysis（Functional connectivity/whole brain analysis）	Exploring the correlation between IPD brain activity and behavior	
Aimee Martin, Stefanie I. Becker and Alan J. Pegna	2021	*	*		*	*	*			Fair
Claudia Massaccesi (et al.)	2021	*		*	*	**	*	*	*	Good
Nasiriavanaki, Z.(et al.)	2021	*	*	*	*	**	*			Good

In line with this tool: “poor”: 1-3 *; “fair”: 4-6 *; 7-9 *: “go

Supplementary 2: Abbreviations

interpersonal distance	IPD
functional magnetic resonance imaging	fMRI
event-related potential	ERP
electroencephalograph	EEG
ventral intraparietal area	VIP
Social anxiety	SA
precentral gyrus	PZ
medial prefrontal cortex	mPFC
orbitofrontal cortex	OFC
dorsal intraparietal sulcus	DIPS
ventral premotor cortex	PMv
Newcastle-Ottawa Quality Assessment Scale	NOS
randomized controlled trial	RCT
comfortable interpersonal distance	CID
prefrontal cortex	PFC
medial prefrontal cortex	mPFC
ventromedial prefrontal cortex	vmPFC
basolateral amygdala	BLA
Autistic Spectrum Disorder	ASD
Borderline Personality Disorder	BPD
Face Fusiform Area	FFA
N2 posterior contralateral	N2pc
amydala	AMY
region of interest	ROI
dorsolateral prefrontal cortex	DLPFC
superior parietal lobules	SPL
anterior insula	AI
dorsomedial prefrontal cortex	dmPFC
Inferior frontal gyrus	IFG
Inferior parietal lobule	IPL
Ventrolateral prefrontal cortex	vlPFC
Psychophysiological interaction	PPI
Temporoparietal junction	TPJ
midbrain periaqueductal grey	PAG
superior frontal cortex	SFC
medial parietal cortex	MPC
superior parietal cortex	SFC
